# Population genomics of an exceptional hybridogenetic system of *Pelophylax* water frogs

**DOI:** 10.1186/s12862-019-1482-4

**Published:** 2019-08-05

**Authors:** Sylvain Dubey, Tiziano Maddalena, Laura Bonny, Daniel L. Jeffries, Christophe Dufresnes

**Affiliations:** 1 Hintermann & Weber SA, Avenue des Alpes 25, 1820 Montreux, Switzerland; 2 Department of Ecology and Evolution, University of Lausanne, Biophore Building, 1015 Lausanne, Switzerland; 3 Agrosustain SA, c/o Agroscope, Route de Duillier 60, 1260 Nyon, Switzerland; 4 Maddalena & associati sagl, 6672 Gordevio, TI Switzerland

**Keywords:** Amphibians, Hybrid speciation, Hybrid amphigamy, Hybrid amphispermy, *Pelophylax* kl. *esculentus*, *P. lessonae*, Polyploidization, RAD-sequencing, Sex chromosomes, Water frogs

## Abstract

**Background:**

Hybridogenesis can represent the first stage towards hybrid speciation where the hybrid taxon eventually weans off its parental species. In hybridogenetic water frogs, the hybrid *Pelophylax* kl. *esculentus* (genomes RL) usually eliminates one genome from its germline and relies on its parental species *P. lessonae* (genomes LL) or *P. ridibundus* (genomes RR) to perpetuate in so-called L-E and R-E systems. But not exclusively: some all-hybrid populations (E-E system) bypass the need for their parental species and fulfill their sexual cycle via triploid hybrid frogs. Genetic surveys are essential to understand the great diversity of these hybridogenetic dynamics and their evolution. Here we conducted such study using RAD-sequencing on *Pelophylax* from southern Switzerland (Ticino), a geographically-isolated region featuring different assemblages of parental *P. lessonae* and hybrid *P.* kl. *esculentus*.

**Results:**

We found two types of hybridogenetic systems in Ticino: an L-E system in northern populations and a presumably all-hybrid E-E system in the closely-related southern populations, where *P. lessonae* was not detected. In the latter, we did not find evidence for triploid individuals from the population genomic data, but identified a few *P. ridibundus* (RR) as offspring from interhybrid crosses (LR × LR).

**Conclusions:**

Assuming *P. lessonae* is truly absent from southern Ticino, the putative maintenance of all-hybrid populations without triploid individuals would require an unusual lability of genome elimination, namely that *P.* kl. *esculentus* from both sexes are capable of producing gametes with either L or R genomes. This could be achieved by the co-existence of L- and R- eliminating lineages or by “hybrid amphigamy”, i. e. males and females producing sperm and eggs among which both genomes are represented. These hypotheses imply that polyploidy is not the exclusive evolutionary pathway for hybrids to become reproductively independent, and challenge the classical view that hybridogenetic taxa are necessarily sexual parasites.

**Electronic supplementary material:**

The online version of this article (10.1186/s12862-019-1482-4) contains supplementary material, which is available to authorized users.

## Background

Hybridization can promote adaptive divergence and speciation [1–4], but interspecific hybrids must first overcome the meiotic disorders associated with gametogenesis of diverged, non-coadapted genomes. The best-studied evolutionary strategies to bypass this barrier include clonal reproduction by parthenogenesis, gynogenesis/hybridogenesis [5] or allopolyploidy, by the production of unreduced diploid gametes [6, 7]. If they are accompanied by reproductive isolation with the parental taxa, these mechanisms can represent the first stages towards hybrid speciation [8, 9]. Nevertheless, hybrid taxa may still arise without clonality and polyploidization [1], and their contribution to biodiversity is presumed to be marginal [10]. Characterizing the processes responsible for the maintenance of hybrid taxa is thus a fundamental step towards understanding how they can lead to speciation.

Hybridogenesis and polyploidization are well-known attributes of *Pelophylax* water frogs [11]. The edible frog *P.* kl. *esculentus* is the hybrid between the pool frog *P. lessonae* (genomes LL) and the marsh frog *P. ridibundus* (genomes RR). It is often found in diploid form (LR) co-existing with one or both of the other parental species. In the *lessonae-esculentus* system (L-E), common in Western Europe, LR hybrids exclude their L genome and produce clonal R gametes. Inter-hybrid crosses yield unviable RR offspring because the R hemiclone irreversibly accumulated deleterious mutations through Müller’s ratchet [12–14]. *Pelophylax.* kl. *esculentus* must then backcross with LL *P. lessonae* to perpetuate, and is therefore considered a sexual parasite. In Eastern Europe, the system is essentially the reverse (*ridibundus-esculentus*, R-E): LR *P.* kl. *esculentus* hybrids predominantly produce L gametes and rely on RR *P. ridibundus* to reproduce.

Interestingly, *P.* kl. *esculentus* can also be widely found by itself in so-called all-hybrid populations (E-E system), where the life cycle is fulfilled by the production of polyploids. Female hybrids can produce bivalent LR eggs that develop into triploid LLR and RRL frogs upon fertilization by haploid L or R hemiclonal sperm, respectively [15–17]. These triploids may also produce unreduced gametes (e. g. LL sperm from LLR males, [17]). As the diploid genomes (LL or RR) are able to recombine in triploids, the all-hybrid population as a whole becomes a sexually functional unit [18]. Yet it is worth noting the strong mutation load of such system, which induces RR and LL zygotes that do not reach sexual maturity [15]. Hence the respective proportions of each gamete (LR, LL, R, L) produced by each sex of each hybrid type (LR, LLR, LRR) is key to the persistence of all-hybrid *P.* kl. *esculentus* populations at an evolutionary stable but sensitive equilibrium [19].

Comparative population genetics of *P.* kl. *esculentus* can shed light on the origin, composition and evolutionary dynamics of all-hybrid populations [20, 21]. Because of the high diversity of breeding systems, clonal genomes, sex-determination and genetic variation in this frog complex [21, 22], comparative analyses of closely-related groups of populations are of prime interest, since their biogeographic history should not be a confounding factor. In addition, many European populations have been largely compromised by multiple invasions of *Pelophylax* alien species, resulting in genetic pollution and/or disruption of their hybridogenetic complexes [23–26].

The present study focusses on *Pelophylax* populations from southern Switzerland, namely the canton of Ticino. This area is mostly inhabited by *P. lessonae* and *P.* kl. *esculentus* in its northern parts (L-E system) but *P. lessonae* frogs are missing from most of the southern parts, which may consist only of E-E systems [12, 27]. This set of populations could therefore provide a standardized framework in which to examine the composition and dynamics of L-E derived all-hybrid *Pelophylax* populations – *P. ridibundus* is naturally absent from the Apennine Peninsula, [28]. The Ticino area also has the advantage of being free of alien *Pelophylax* taxa [24].

South-alpine populations are also attractive from a phylogenetic perspective. Our recent phylogeography of water frogs from the Apennine Peninsula revealed a cryptic nuclear lineage basal to the two known pool frog taxa (*P. lessonae* and *P. bergeri*), and restricted to the Alpine catchments valleys of the Po plain (named “*Pelophylax.* n. t. 2”, [29]). Mostly based on the intronic sequence marker *Serum Albumin* intron 1 (SAI-1), but lacking mitochondrial divergence, the origin of this lineage is pending additional analyses.

In order to characterize the genetic nature and hybridogenetic mechanisms of the subalpine *Pelophylax* populations, we conducted a population genomic and morphometric survey of nine sites inhabited by *P. lessonae* and *P.* kl. *esculentus* in southern Switzerland. The objectives were (1) to assess the genetic composition of putative L-E and E-E populations, (2) to understand whether E-E populations are maintained through triploid individuals or other mechanisms, and (3) to infer the nature and origin of the *P.* n. t. 2 lineage previously proposed [29].

## Results

### Population genomics of Pelophylax in Ticino

**Fig. 1:**
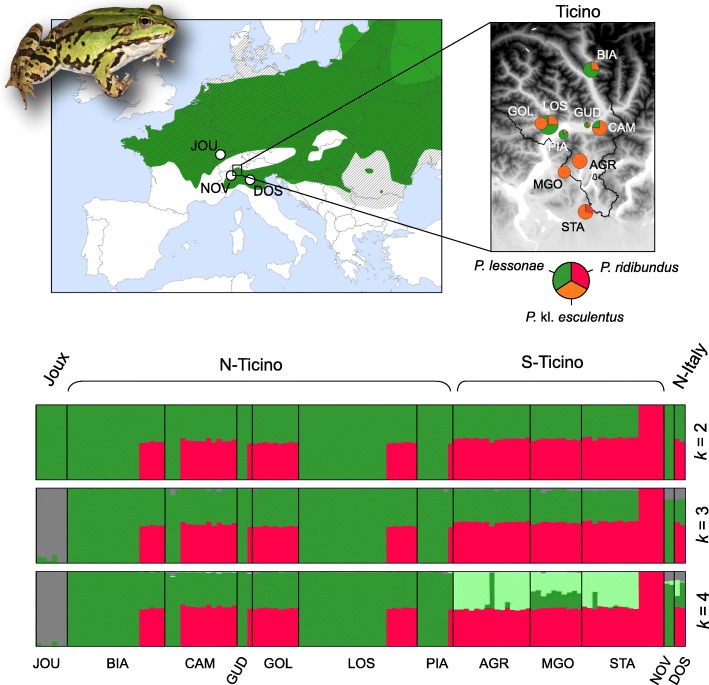
Location of the study area and reference samples, proportion of each species sampled in each population of Ticino, and individual ancestry to the *P. lessonae* (green/grey) and *P. ridibundus* (red) gene pools, as inferred by STRUCTURE with *k* = 2–4 (based on 2521 SNPs). The hybrid frogs *P.* kl. *esculentus* accordingly received intermediate ancestry values. Three *P. lessonae* gene pools were identified, including two in Ticino. The distributions of *P. lessonae* (plain green) and *P.* kl. *esculentus* (dashed lines) are shown on the map, based on the IUCN red list data. Photo: *P.* kl. *esculentus* from AGR (diploid all-hybrid population); photo and map credit: C. Dufresnes

In northern Ticino, we sampled both *P. lessonae* and *P.* kl. *esculentus*, occurring in syntopy at most sites (Fig. 1, Table 1). In southern Ticino, we only found *P.* kl. *esculentus* from the three extant water frog populations known, expect a few hundred meters from site STA, where we captured 5 females of the *P. ridibundus* morphotype (Fig. 1, Additional file 2: Table S1).

**Table 1:** Number of frogs caught (*n*) and analyzed with RAD, mtDNA, L/R diagnostic microsatellites (μsat) and morphometrics (morpho) in Ticino populations; lat.: latitude, long.: longitude; H_o_: observed heterozygosity

Code	Locality	lat.	long.	Species	*n*	RAD	mtDNA	μsat	morpho	H_o_
*Northern Ticino*										
BIA	Biasca	46.39	8.98	*P. lessonae*	14	14	10	14	14	0.06
				*P.* kl. *esculentus*	5	5	2	5	5	0.21
CAM	Camorino	46.16	9.01	*P. lessonae*	4	3	3	4	4	–
				*P.* kl. *esculentus*	13	11	9	13	13	0.19
GUD	Gudo	46.17	8.95	*P. lessonae*	2	2	2	–	–	–
				*P.* kl. *esculentus*	1	1	1	–	–	–
GOL	Golino River	46.18	8.71	*P.* kl. *esculentus*	12	9	9	12	8	0.18
LOS	Losone	46.17	8.74	*P. lessonae*	22	17	16	20	20	0.07
				*P.* kl. *esculentus*	7	6	6	3	3	0.19
PIA	Piazzogna	46.14	8.82	*P. lessonae*	6	6	6	–	–	0.07
				*P.* kl. *esculentus*	1	1	1	–	–	–
*Southern Ticino*										
AGR	Agra	46.03	8.90	*P.* kl. *esculentus*	17	15	15	16	15	0.21
MGO	Monteggio	45.99	8.81	*P.* kl. *esculentus*	12	10	11	11	12	0.21
STA	Stabio	45.84	8.91	*P.* kl. *esculentus*	12	11	12	11	12	0.21
				*P. ridibundus*	5	5	5	5	5	0.02

**Fig. 2:**
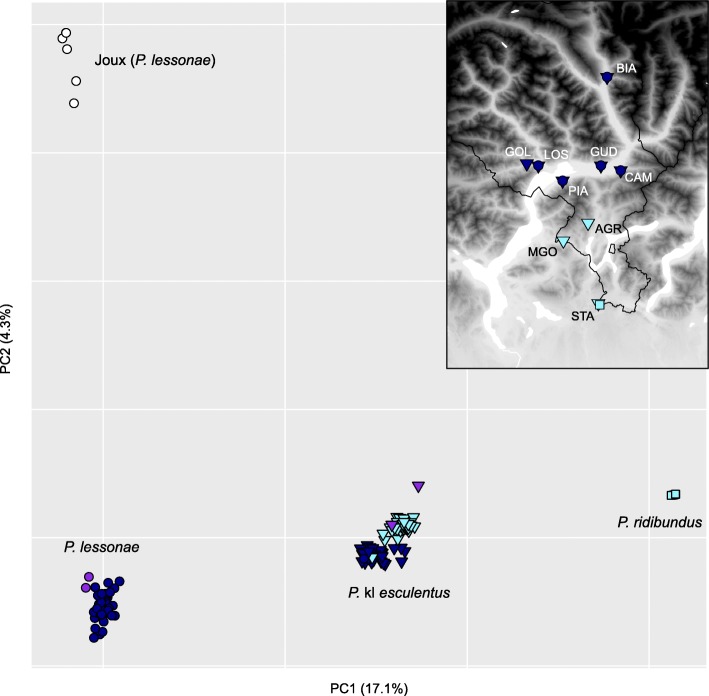
PCA on individual genotypes (2521 SNPs) from *P. lessonae* (circles), *P.* kl. *esculentus* (triangles) and *P. ridibundus* (squares) sampled in northern (dark blue) and southern Ticino (light blue), as well as nearby reference populations (purple: northern Italy; white: Joux Valley in W-Switzerland)

Genetic analyses corroborated our field observations. Based on 2521 SNPs, Bayesian clustering with STRUCTURE (*k* = 2) recovered the two main gene pools corresponding to *P. lessonae* (northern Ticino, Joux Valley, northern Italy) and *P. ridibundus* (STA). Hybrids *P.* kl. *esculentus* were accordingly assigned with half probabilities (Fig. 1). Analyses with increasing *k* separated *P. lessonae* from the north-alpine Joux Valley (*k* = 3) and distinguished the L genomes of southern Ticino hybrids (*k* = 4). The most likely *k* was *k* = 2 according to the *Δk* statistic (*Δk* = 3689.9) and *k* = 3 according to the *L(k)* statistic (*L(k)* = − 108,408.6). In south Ticino, one *P.* kl. *esculentus* (AGR08) featured the northern Ticino L genome, while all others had the southern Ticino L, or a mix of both (Fig. 1). The first axis of the PCA depicted a similar signal, highlighting the genetic structure within *P. lessonae* and between *P.* kl. *esculentus* from north and south Ticino (Fig. 2). Genetic diversity was accordingly higher for the hybrid *P.* kl. *esculentus* (H_o_ = 0.18–0.21) compared to the parental *P. lessonae* (H_o_ = 0.06–0.07) (Table 1, Additional file 1: Figure S1). The five *P. ridibundus* specimens featured very low heterozygosity (H_o_ = 0.02) (Table 1, Additional file 1: Figure S1).

**Fig. 3:**
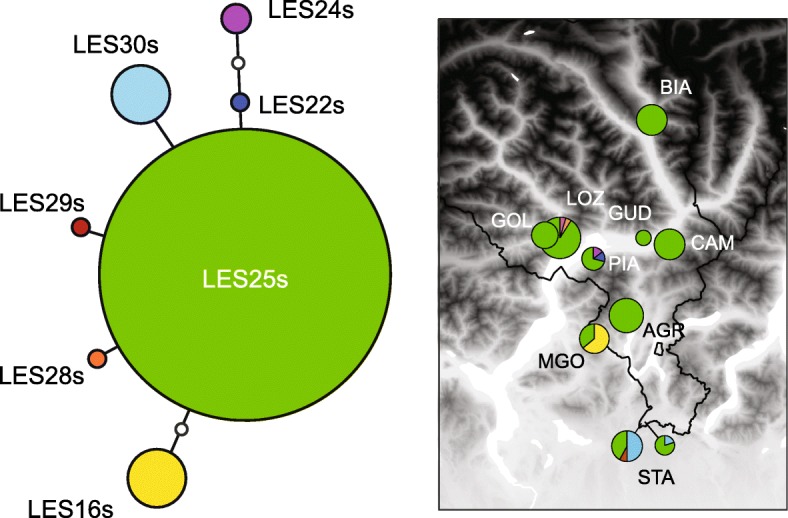
Haplotype network and geographic distribution of the mitochondrial *cyt-b* haplotypes sampled in Ticino. All belong to *P. lessonae*. Haplotype numbers correspond to the ~ 900 bp sequences published by Dufresnes et al. [24] but are labelled “s” for “short” since here only ~ 500 bp was sequenced

All frogs from Ticino possessed *P. lessonae* mtDNA (Fig. 3). A single *cyt-b* haplotype (LES25s) was sequenced in all populations but a few additional ones were private from northern (LES22s, LES24s and LES28s) and southern Ticino (LES16s, LES30s). The *P. ridibundus* females from STA featured two different *P. lessonae* haplotypes (LES25s and LES30s).

### Phylogenomics of Pelophylax in Ticino

**Fig. 4:**
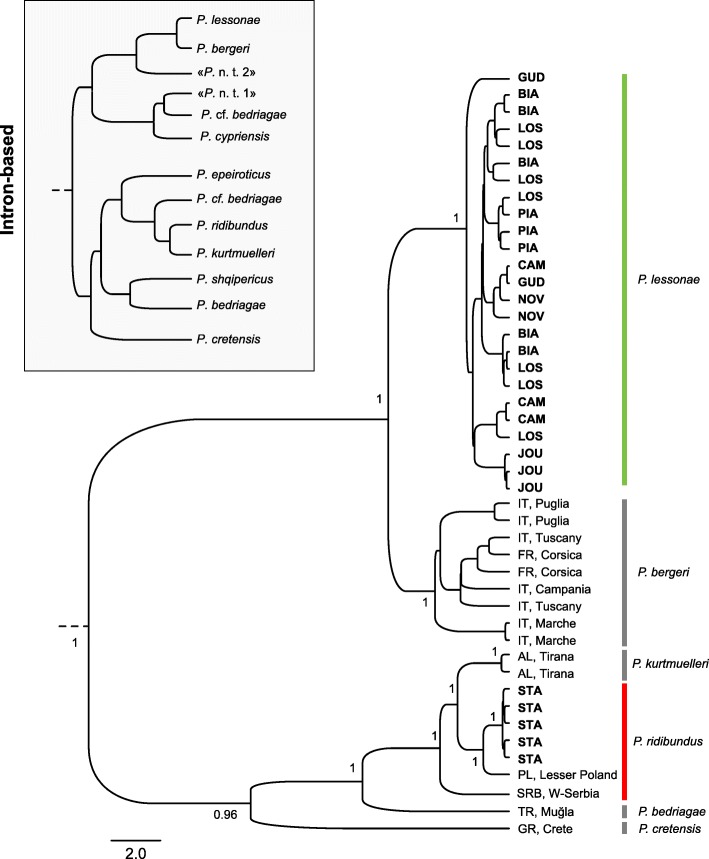
Bayesian phylogenetic reconstruction of 13.1 kb of genome-wide nuclear data (RAD tags) for non-hybrid water frogs from Ticino (in bold) and reference samples. All south-alpine frogs belong to the *P. lessonae* clade, expect for the five *P. ridibundus* females collected at STA, which branch to the corresponding *P. ridibundus* clade. The intron-based phylogeny of Dubey & Dufresnes [29] is provided for comparison (~ 1.6 kb from two markers, including 1.4 kb from SAI-1); “*P.* n. t. 1”: hemiclone sequenced in the Italian hybridogens *P.* kl. *hispanicus*; “*P.* n. t. 2”: putative endemic lineage sequenced in south-alpine *P. lessonae* and *P.* kl. *esculentus*. Our genomic data clearly rule out the existence of the latter. Country codes used for the reference samples as follows. AL: Albania; FR: France; GR: Greece; IT: Italy; PL: Poland; SRB: Serbia; TR: Turkey

Phylogenetic reconstruction of RAD sequences (13.1 kb) recovered the six species included in the analysis (Fig. 4). Parental *Pelophylax* frogs from northern Ticino all belong to a fully supported monophyletic *P. lessonae* clade, sister of *P. bergeri*, with little intraspecific structure. The five frogs from southern Ticino identified as *P. ridibundus* were accordingly grouped with our *P. ridibundus* reference samples.

### Identification of triploids

**Fig. 5:**
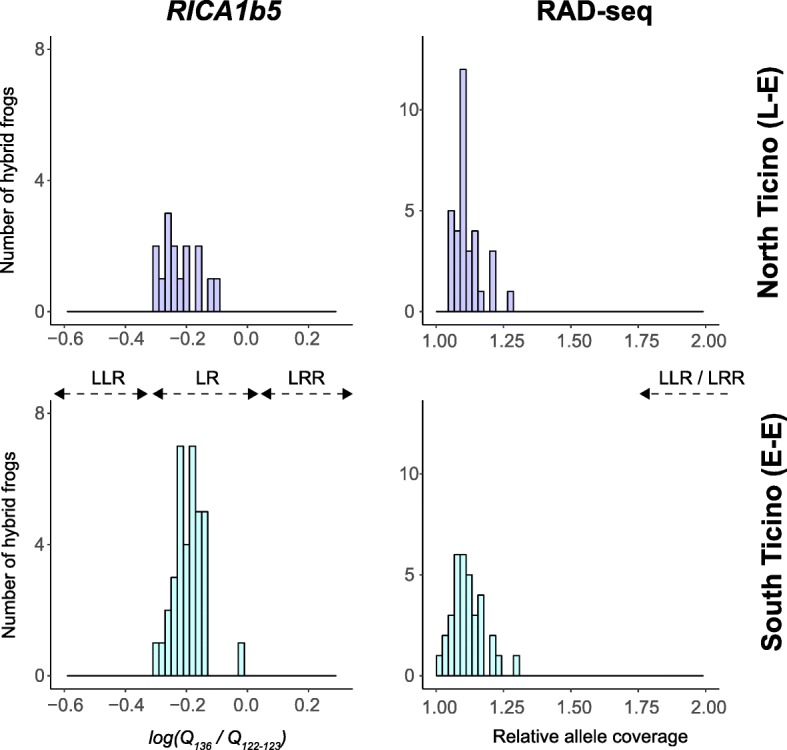
Left: ratio of allele quantities as *log(Q*_*R*_*/Q*_*L*_*)* for the hybrid genotypes *122*/*137* and *123*/*137* in northern Ticino (top panel) and southern Ticino (low panel). All fall within the range of LR diploid frogs bearing the analogous genotype *120/136* analyzed by Christiansen [30]. Ratio obtained for triploids LLR and LRR of the same genotype are provided for comparison. Note that given the high diversity of L-specific alleles (see Results), we would have also detected LLR triploids as tri-allelic individuals. Right: average differences in coverage between the L/R diagnostic alleles of each *P.* kl. *esculentus* frog sampled in northern and southern Ticino, based on the RAD data

We could not find evidence of triploid hybrid frogs in Ticino. No tri-allelic genotypes were found at the three diagnostic L/R microsatellite loci: *Res16*, *Rica1b5* and *Rica2a34*, see Methods). For locus *Res16*, allele *127* was fixed in the R genome, while five different alleles segregate on the L genome, including allele *127* and a null allele (Additional file 2: Table S1). For locus *Rica2a34*, allele *106* and a null allele could be isolated from the R genome, while 16 other variants segregate on the L genome (Additional file 2: Table S1). Because of this strong variation, both in terms of polymorphism and amplification success, it was not appropriate to quantify the peak height ratio (PHR) between the two alleles of hybrid frogs for *Res16* and *Rica2a34*. For locus *Rica1b5*, allele *137* was R-specific in all populations, while alleles *122*, *123*, *127* and *145* were L-specific (Additional file 2: Table S1). Fortunately, the majority of *P.* kl. *esculentus* frogs were represented by the nearly-identical genotypic profiles *122*/*137* (*n* = 31) and *123*/*137* (*n* = 20), which allowed for comparison of their PHR. Computed as *log(H*_*137*_ */H*_*122–123*_*)* (see Methods), the PHR averaged − 0.22 (− 0.29 – − 0.09) in northern Ticino (putative L-E system) and − 0.19 (− 0.31 – − 0.03) in southern Ticino (putative E-E system, Fig. 5), which fall within the range obtained by Christiansen [30] for diploid LR frogs of an analogous genotype at this marker (*120*/*136*; PHR = − 0.23 – 0.00). In contrast, Christiansen [30] reported PHR averaging − 0.39 (− 0.54 – − 0.29) for LLR triploids and 0.17 (0.09–0.25) for LRR triploids at this genotype (illustrated in Fig. 5 for comparison).

RAD markers also supported diploidy for all hybrid frogs. Among the 2521 SNP genotypes, we identified 376 RAD tags with fixed differences between *P. lessonae* and *P. ridibundus*. For these L/R diagnostic loci, relative allele coverage (highest allele coverage/lowest allele coverage) tended towards 1 for all loci (mean = 1.1×, range: 1.01× − 1.3×). This average value (1.1×) was also obtained separately for northern (putative L-E system) and southern Ticino (putative E-E system) (Fig. 5). No frogs showed relative coverage ratios anywhere near 2×, as expected for LLR or LRR triploids.

### Morphometric analyses

**Fig. 6:**
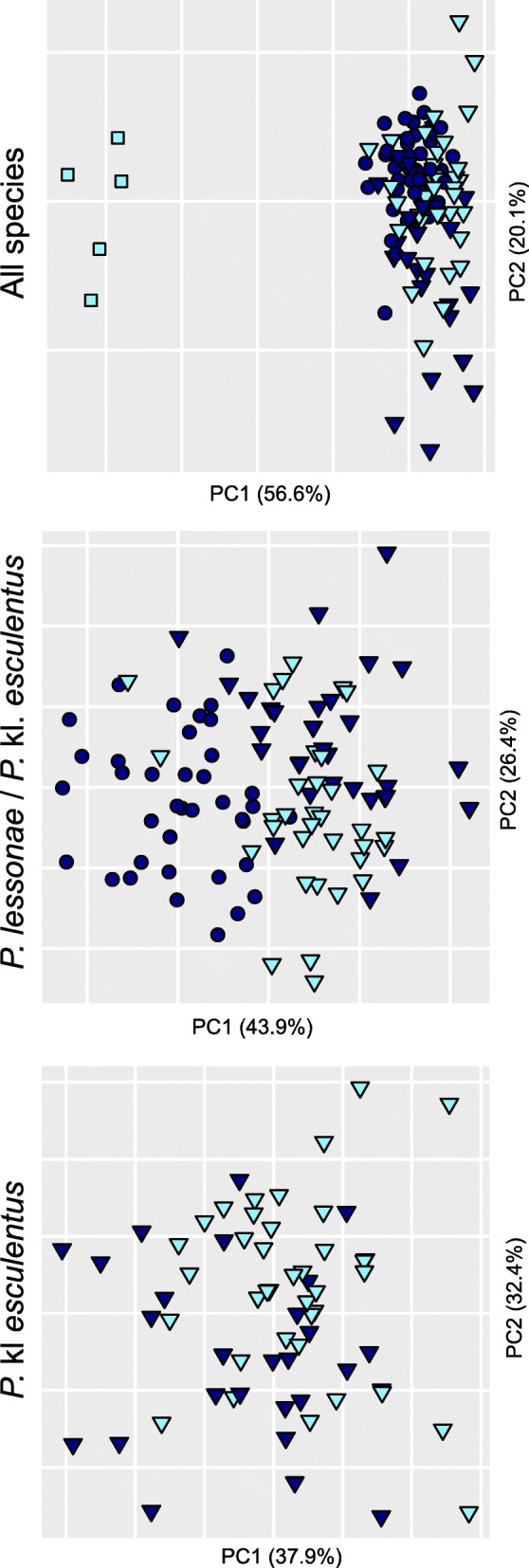
PCA on morphometric data combining all three species found in Ticino (top), *P. lessonae* with *P.* kl. *esculentus* (middle), and *P.* kl. *esculentus* only (bottom). Color and symbols discriminate geographic origin (dark blue: northern Ticino; light blue: southern Ticino) and species (squares: *P. ridibundus*; circles: *P. lessonae*; triangles: *P.* kl. *esculentus*)

A MANOVA analysis on Ticino frogs combining five morphometric variables (see Methods) suggested a significant effect of taxa (*F* = 54.1, *P* < 0.001) and population (*F* = 2.6, *P* < 0.001), but not of sex (*F* = 1.9, *P* = 0.10). PCAs on different sets of individuals (both sexes pooled) clearly differentiated the five *P. ridibundus* of site STA (Fig. 6, top), as well as between *P. lessonae* and *P.* kl. *esculentus* (Fig. 6, middle). The difference between northern and southern *P.* kl. *esculentus* was not obvious (Fig. 6, bottom), but remained significant even when including sex and population in the MANOVA (*F* = 4.7, *P* = 0.002).

## Discussion

### Two contrasting hybridogenetic systems in Ticino

Several hybridogenetic systems are known from the *Pelophylax* model, where the hybrid *P.* kl. *esculentus* co-exists with either *P. lessonae* (L-E system), *P. ridibundus* (R-E system), sometimes with both (L-R-E system) and sometimes with neither (E-E system), in which case the sexual cycle relies on triploid individuals (see Background). In Ticino, we characterized two putatively different hybridogenetic systems in otherwise closely-related populations.

**Fig. 7:**
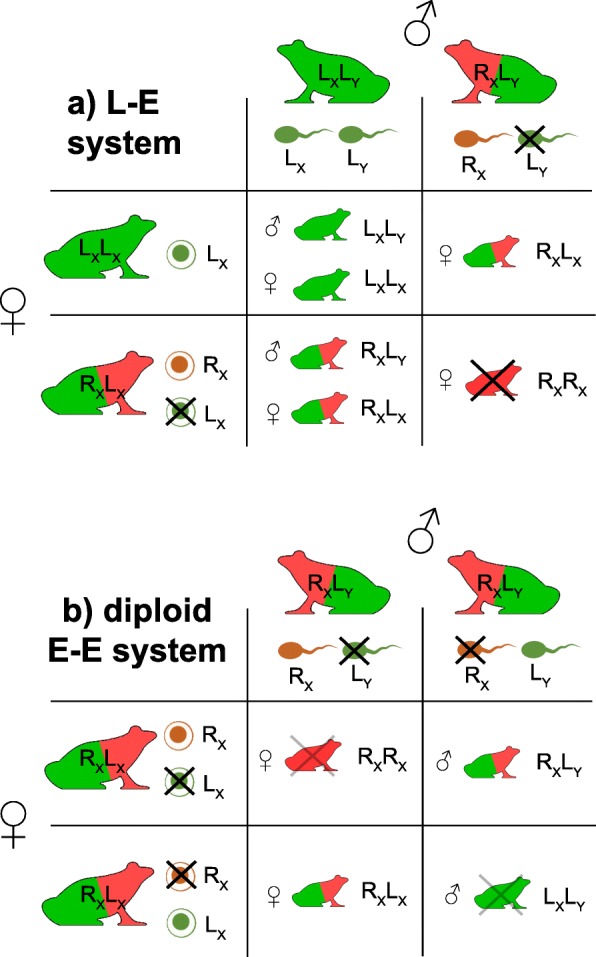
Outcomes of crosses for the putative hybridogenetic systems proposed to inhabit Ticino. Northern populations correspond to the classic L-E system (**a**). In southern Ticino, the hypothetical maintenance of LR hybrid frogs without triploids and without *P. lessonae* requires that frogs from both sexes alternatively eliminate the L and the R genomes (**b**). RR and LL genotypes are supposedly unfit (hybridogenetic load) but can arise in populations, as we found in STA

In northern Ticino, the hybrid *P.* kl. *esculentus* was found together with *P. lessonae* at sites BIA, CAM, GUD, PIA and LOS. At GOL (a river bank) only hybrids were captured but these frogs most likely breed elsewhere, perhaps at the nearby site LOS, where *P. lessonae* occurs in large numbers. Hence, all these populations fit the expectations of an L-E system, where *P.* kl. *esculentus* exclusively produces R gametes and backcrosses *P. lessonae* to perpetuate (Fig. 7a).

In southern Ticino however, we did not find *P. lessonae* at any site, despite equivalent search efforts. All populations were exclusively composed of *P.* kl. *esculentus* of both sexes, except for five females at site STA that we unexpectedly identified as *P. ridibundus* (Table 1). Several clues indicate that these *P. ridibundus* were not parental frogs, but rather the offspring of *P.* kl. *esculentus* × *P.* kl. *esculentus* hybrid crosses. First, *P. ridibundus* is not naturally present in Ticino and northern Italy; the closest naturally-connected populations are in Croatia [28]. Second, all five possessed local *P. lessonae* mitotypes, while parental *P. ridibundus* normally conserve their maternal lineages throughout hybridogenesis, because of mating preferences: in mixed populations, the large *P. ridibundus* females are preferentially chosen by the males of the other smaller species, rarely the other way around (e.g. [26]). Third, the nuclear diversity of these females was extremely low (H_o_ = 0.02), as expected for hemiclonal RR individuals. Fourth, these frogs were much smaller (SVL = 45–65 mm) than regular *P. ridibundus* (up to 170 mm, [28]). While they could be subadults, all our other observations at the same time of the year in Ticino involved sexually mature frogs, and their small size could rather reflect the mutation load and low fitness expressed by diploid clonal RR genotypes. Although rarely fit, non-hybrid frogs are supposed to arise every year in all-hybrid populations [15, 31]. Hence, southern Ticino could be inhabited by all-hybrid E-E- systems that sometimes produce non-hybrid individuals.

Surprisingly however, we did not find evidence for mixed ploidy. First, L/R-diagnostic SNP alleles received even sequence coverage in all hybrids, as expected for LR diploids, where the L and R alleles should be present in equal quantities. While no confirmed triploid frogs could be included here as controls, the same approach efficiently disentangles diploids from asymmetric polyploids in other hybridogenetic systems (G Lavanchy, pers. com.). Second, no individual was tri-allelic at our diagnostic microsatellites. For the latter, the strong diversity of L alleles should have allowed to detect LLR individuals if these were present. Third, the allelic profiles of our hybrid frogs were far from the range of allele quantity difference reported for LRR frogs at the microsatellite locus *RICA1b5* [30]. Hence, the data at hand suggests that the putatively all-hybrid *P.* kl. *esculentus* populations of southern Ticino are maintained without triploids, unlike in other parts of Europe. This assumption necessitates confirmation by direct evidence from experimental crosses to trace allele inheritance, and from cytogenetics.

Alternatively, *P. lessonae* could be cryptically present in southern Ticino, in which case populations would be composed of unnoticed L-E systems. In a bioacoustic survey over 2001–2002, Mattei-Roesli & Maddalena [27] mostly reported *P.* kl. *esculentus* in this area, but suspected the presence of *P. lessonae* at a few sites. A few years before, Vorburger [12] identified two *P. lessonae* among tens of *P.* kl. *esculentus* captured nearby STA (locality Seseglio, now extinct). These observations could represent a recent shift in the composition of these populations (as seen elsewhere [21]), but they could also be the scarce LL offspring from hybrid crosses (rather than parental *P. lessonae*). Occasional dispersal from northern to southern Ticino is also possible, as illustrated by one “northern” frog caught at site AGR (Fig. 2). Therefore, formally rejecting the hypothetical presence of breeding *P. lessonae* in southern Ticino will require additional monitoring efforts throughout an entire breeding season.

### On the causes and consequences of a putative diploid all-hybrid system

How could a diploid all-hybrid E-E hybridogenetic system perpetuate? To our knowledge, such situation has never been reported. Importantly, because sex is supposedly determined by an XY system [32], and because primary hybridization events preferentially involve *P. lessonae* males (L_x_L_y_) with *P. ridibundus* females (R_x_R_x_), the L hemiclone of *P.* kl. *esculentus* hybrids can carry either an X or a Y, while the R is strictly X-linked, i. e. hybrid males are L_y_R_x_ and hybrids females are L_x_R_x_ [33]. Therefore, both sexes must provide L and R gametes so sons and daughters can be generated (Fig. 7b). As a consequence, R_x_R_x_ females (like the ones we found in STA, see also [31]) and L_x_L_y_ males should also be produced. However, it is worth noting that Vorburger [12] failed to obtain any viable metamorphs from a few inter-hybrid crosses from the extinct Seseglio site. This suggests an important hybridogenetic load and if they exist, that hybrid frogs producing both L and R gametes might be rather infrequent. In counterpart, the occasional LL and RR individuals would provide opportunities for recombination to purge deleterious mutations from hemiclones.

The pre-requisites of this putative diploid E-E system are difficult to reconcile given our current knowledge of *Pelophylax* gametogenesis. In R-E populations, *P.* kl. *esculentus* males can produce sperm of either hemiclones [34], or sometimes both simultaneously by hybrid amphispermy [35, 36]. We are not aware of reciprocal dynamics in female hybridogens, which either transmit their R hemiclone (in L-E systems), or both the R and L within diploid eggs (in regular E-E- systems with mixed ploidy) [34]. Alternated L or R genome exclusion between hybrid females, or between the germ cells of the same female – what could be referred to as “hybrid amphigamy” – is yet to be documented. Nevertheless, gametogenesis and notably oogenesis might be more labile than previously assumed in diploid *P.* kl. *esculentus* [34], including the mechanism and timing of genome exclusion [37].

How could such a system arise? Lability in genome elimination could stem from a mixed origin of these frogs, involving secondary contact between L- and R-eliminating lineages. The amphispermic hybrid reported by Ragghianti et al. [36] was a cross between *P. lessonae* from a L-E system and *P. ridibundus* from a R-E system. Water frogs most likely colonized the south-alpine region from a Central European refugia, where a great diversity of hybridogenetic systems (including L-E and R-E populations) and clonal lineages co-exist [21].

The effective maintenance of a diploid *P.* kl. *esculentus* system, although pending further investigations to confirm the total absence of *P. lessonae* and of triploids, contributes to the ongoing debate of the evolutionary fate of hybridogenetic hybrids. First, since both genomes would be transmitted, this system challenges the usual view that hybridogenetic hybrids are sexual parasites [38]. Second, it suggests that polyploidization is not required to become reproductively independent, as a preliminary stage of hybrid speciation. Triploidy is often seen as a springboard towards tetraploidy, from which hybrid species are easier to evolve [39]. The diploid hybridogens from Ticino would emphasize an alternative pathway, although whether they are truly self-sustainable (if they reproduce by “hybrid amphigamy”) or rely on interdependent L- and R-eliminating lineages, remains an open question. In a later step, these populations could eventually evolve reproductive isolation from *P. lessonae* and *P. ridibundus* by allopatric divergence, leading to homoploid hybrid speciation. Such outcome yet appears unlikely given the frequent reshuffling of amphibian distributions throughout the Quaternary. For the time being, water frogs from Ticino represent some of the last genuine *Pelophylax* assemblages in Western Europe and offer a promising framework to study these fascinating aspects of hybridogenesis.

### No evidence for a south-alpine endemic water frog lineage

In contrast to our previous investigations based on the SAI-1 intronic marker [29], we did not recover a south-alpine lineage endemic to Ticino and northern Italy using genome-wide RAD data. Instead, all pool frogs belonged to a monophyletic, well-supported *P. lessonae* clade (Fig. 4) and all possessed *P. lessonae* mitotypes (Fig. 3). SAI-1 thus features strong ancestral polymorphism and does not always seem representative of the evolutionary history of species, perhaps because it contains a retro-transposon [40]. Hence, we recommend that phylogenetic and phylogeographic inferences relying on this marker to be treated with caution (e.g. [29, 41, 42]).

In particular, we previously hypothesized that the hemiclone of Italian hybrid frogs *P.* kl. *hispanicus* is related to an undescribed extinct lineage of Anatolian origin, based on SAI-1 variation (“*P.* n. t. 1”, clearly differing from the north-Italian hemiclones; [29]). Alternatively, this "lineage" could thus simply represent intraspecific SAI-1 alleles of *P. ridibundus*, or of a related Middle Eastern taxon. Yet, despite several molecular surveys focusing on this region [41, 42], such alleles have still never been reported from extant populations. Similarly, the Cyprus endemic *P. cypriensi*s, which was described from mtDNA and nuclear SAI-1 divergence [42], deserves a re-evaluation. *Pelophylax* phylogeography and systematics are in clear need for more comprehensive molecular analyses, as offered by genome-wide loci.

## Conclusions

Through a comprehensive genomic survey, we rejected the hypothesis of an endemic south-alpine lineage of *Pelophylax* water frogs, and emphasized two types of hybridogenetic systems from southern Switzerland: a rather classic *P. lessonae* – *P.* kl. *esculentus* (L-E) system in northern populations and a putatively all-hybrid *P.* kl. *esculentus* (E-E) system in the south, where frogs unexpectedly showed no sign of triploidy. Nevertheless, we cannot formally exclude the cryptic presence of *P. lessonae* in the latter, and call for future monitoring efforts in southern Ticino and nearby Italy. If confirmed, these all-hybrid diploids could persist by labile genome elimination, i. e. frogs produce eggs and sperm carrying L or R genomes indiscriminately, which would challenge the classic views that hybridogenetic hybrids are sexual parasites and that they require transient polyploid steps to reach sexual independence.

## Methods

### Sampling

In July 2017 and 2018, nine localities were surveyed by day and night under good meteorological conditions for *Pelophylax* activity (sunny days, temperatures between 20 °C and 30 °C), covering the entire distribution of these frogs in the canton of Ticino (southern Switzerland) (Table 1). A total of 115 individuals were captured and identified based on the shape of their metatarsal tubercle [28]. Buccal cells were sampled using non-invasive cotton swabs and adults (*n* = 111) were measured for the following variables, relevant for comparative morphometry in *Pelophylax*: snout-vent length (SVL), tibia length (LTi), total hind leg length (LTo), length (LMT) and height (HMT) of the metatarsal tubercle. Animals were sampled and measured directly in the field, and then immediately released at their place of capture.

DNA was extracted using the Qiagen BioSprint Robotic workstation. In complement, we included 18 DNA samples collected in the study area during Spring 2014, as well as 25 samples from other *Pelophylax* populations/species available from our previous studies [24, 29], used as references to identify the taxa inhabiting Ticino. The latter consisted of six *P. lessonae* from the Joux Valley (north-western Switzerland), four *P. lessonae*/*P.* kl. *esculentus* from northern Italy, nine *P. bergeri* from Italy and Corsica, two *P. ridibundus* from eastern Europe, two *P. kurtmuelleri* from Albania, one *P. cretensis* from Crete, and one *P.* c.f. *bedriagae* from Turkey. Full details are provided in Additional file 2: Table S1.

### mtDNA sequencing

For mtDNA-based barcoding, a portion of *cytochrome-b* (*cyt-b*) was sequenced and aligned (511 bp) in 91 samples from Ticino, using custom Ranid-specific primers CytB-F2 (5′-TTAGTAATAGCCACAGCTTTTGTAGGC-3′) and CytB-R2 (5′-AGGGAACGAAGTTTGGAGGTGTGG-3′). Amplification were carried out in 25 μL reaction volumes, including 1 μL of each primers (10 μM), 7.5 μL of Qiagen Multiplex Primer Mix (MPMM, a premix including hot-start polymerase, dNTP and buffer), 12.5 μL of milli-Q water and 3 μL of template DNA. The PCR ran as follow: 95 °C for 15′, 35 cycles of 94 °C for 30″, 53 °C for 45″ and 72 °C for 1′, followed by 10′ at 72 °C. Longer *cyt-b* haplotypes published for 18 additional samples from this region [24] were included in the downstream analyses.

### RAD-sequencing

We prepared a double digest RAD (ddRAD) multiplexed library following the protocol by Brelsford et al. [43], which performs nicely for population genomics in anuran amphibians (e. g. [44]), including *Pelophylax* [45]. The library contained the 133 frogs from Ticino plus the 25 reference samples, and was sequenced on two Illumina lanes (single read 125). Raw sequences were quality-checked (FastQC v0.10.1) and processed with Stacks v1.48 [46] to demultiplex, stack and catalog homologous loci in all samples using the default *-m -n*, and *-M* values. We then called SNPs to conduct population genomic analyses on Ticino and the closely-related frogs from Joux and northern Italy. We flagged 17 Ticino samples featuring high rates of missing data with a custom python script (available at: https://github.com/DanJeffries/RADweek/blob/master/code/Summary_plotter.py), and subsequently outputted a genotype matrix for 126 samples, considering SNPs present in 80% of individuals of each population (2521 SNPs). We also produced a sequence alignment (13,098 bp from 111 RAD tags) for 45 non-hybrid individuals from different *Pelophylax* taxa, to be used in the phylogeny (list in Additional file 2: Table S1).

### Genetic detection of triploid hybrids

We followed two separate approaches to identify the ploidy of hybrids *P.* kl. *esculentus*. First, we genotyped microsatellite loci known to co-amplify and have specific L and R alleles: *RICA1b5*, *Res16* and *RICA2a34* (reviewed in [47]). Triploids may be tri-allelic, or, in the absence of polymorphism on the duplicated genome, one allele should be amplified in double quantity compared to the other. Because the amplification performance of microsatellites is also affected by allele size and potential nucleotide mismatch on the priming sequence, the height of the absorbance peaks must be interpreted with caution and independently for different genotypes. Christiansen [30] calibrated such approach for several northeastern European genotypes and showed that their peak height ratio (PHR) were not overlapping between LLR, LR and LRR hybrids, and could thus be used as an identification tool.

We amplified the three loci in 113 water frogs from Ticino by 10 μL multiplexed PCRs containing 3 μL of MPMM, 2.2 μL of milli-Q water, 3 μL of template DNA, as well as primers (10 μM) for *RICA1b5* (0.1 μL each), *Res16* (0.3 μL each) and *RICA2a34* (0.5 μL) each. PCRs were conducted as follow: 95 °C for 15′, 35 cycles of 94 °C for 30″, 53 °C for 45″ and 72 °C for 1′, followed by 30′ at 60 °C. Amplicons were diluted 4× and run on an ABI Prism 3100 genetic analyzer. Importantly, PCR conditions and dilution were optimized to ensure the readability of absorbance peaks. Peaks were scored and their height measured with GeneMapper 4.0 (Applied Biosystems). When comparable (see Results), we calculated the PHR as *log(H*_*R*_*/H*_*L*_*)*, where *H*_*R*_ is the height of the R-specific allele, and *H*_*L*_ is the height of the L-specific allele, following Christiansen [30].

Our second approach aimed at comparing the coverage (sequence depth) between RAD tags with fixed L-R differences. In LR diploids, the two alleles should have approximately been sequenced at the same depth, and so their relative coverage should on average tend towards one. In LLR and LRR triploids however, one allele should have been sequenced twice compared to the other and so their relative coverage should on average tend towards two. To compute L-R coverage differences, we first flagged SNPs that were fixed between the R and the L genome, i. e. with allele frequency differences of 1.0 between the frogs identified as *P. lessonae* (LL) and *P. ridibundus* (RR) in our study area (see Results). For each of these SNPs, we then calculated the ratio of the highest allele coverage by the lowest, for every *P.* kl. *esculentus* hybrid frog identified in the study area.

### Population genetic analyses

We explored the genetic structure of the water frog populations from Ticino, Joux and northern Italy based on our matrix of 2521 SNPs (*n* = 126 individuals). First, we used the Bayesian clustering algorithm of STRUCTURE [48] with the admixture model and performed three replicate runs from *k* = 1 to 6, each with 100,000 iterations after a burnin period of 10,000. Because the divergence between the L and R genomes (~ 16Mya, G. Mazepa unpublished data) pre-date any intraspecific differentiation, we expected two major gene pools and thus *k* = 2 as the most informative solution, which we verified with STRUCTURE Harvester [49]. Second, we conducted a Principal Component Analyses (PCA) on individual genotypes with the R packages *adegenet* and *ade4*. We also computed average heterozygosity for each population with *n* ≥ 5, separately for *P. lessonae*, *P.* kl. *esculentus* and *P. ridibundus*.

In addition, we visualize mitochondrial sequence variation of our ~ 500 bp *cyt-b* fragment in Ticino by an haplotype network (TCS, [50]).

### Phylogenetic analyses

We conducted a Bayesian phylogenetic reconstruction of RAD sequences of non-hybrid frogs from Ticino, complemented by reference individuals from six different species (*n* = 45, 13.1 kb; Additional file 2: Table S1). This analysis was performed in BEAST (BEAST 2.4.8, [51]). We used a lognormal relaxed molecular clock calibrated to the divergence of the Cretan endemic *P. cretensis* at the end of the Messinian Salinity Crisis at 5.33 ± 1.0 Mya [52], and the midpoint root between pool frogs (here represented by *P. lessonae* and *P. bergeri*) and marsh frogs (here represented by *P. ridibundus*, *P.* cf. *bedriagae*, *P. cretensis* and *P. kurtmuelleri*) at 16 ± 3.0 Mya My (G. Mazepa, unpublished data), using normally distributed priors and a birth-death tree model. We applied a GTR + G substitution model (BEAST package bModelTest; [53]) and ran the chain for 100 million iterations, sampling one tree every 50,000. We verified stationarity and effective sample sizes of parameters with Tracer 1.5, and built maximum-clade credibility trees with the BEAST module TreeAnnotator, discarding the first 20% of sampled trees as burnin.

### Morphometric analyses

The morphology of Ticino frogs was assessed using the field-measured variables LTi, LTo, LMT and HMT corrected by individual size (SVL), as well as the ratio LMT/HMT, which reflects the shape of the metatarsal tubercle (an important anatomical feature to compare *Pelophylax* taxa and their hybrids, [28]). Combining these five variables, the general morphology was first compared between species, sex and population by a MANOVA. Because there were no significant differences between sexes (see Results), we then conducted several PCA analyses (*ade4* package in R) with males and females pooled together.

## Additional files

Additional file 1: **Figure S1.** Average heterozygosity of *Pelophylax* populations mapped. (PDF 1249 kb)

Additional file 2: **Table S1.** Origin and details on the individuals used in this study. (XLSX 26 kb)

## Data Availability

Microsatellite genotypes, *cyt-b* haplotypes and morphometric measurements are provided in Additional file 2: Table S1. *cyt-b* haplotype sequences are available on GenBank (MF094328-MF094354). Raw sequence reads from the RAD libraries are available on the NCBI SRA archive under bioproject PRJNA549908.

## Abbreviations

*cyt-b*: *cytochrome-b*
ddRAD: double digest restriction site associated DNA
DNA: deoxyribonucleic acid
E-E system: *esculentus-esculentus* system
Fig.: figure
HMT: metatarsal tubercle height
L-E system: *lessonae-esculentus* system
LMT: metatarsal tubercle length
L-R-E: *lessonae-ridibundus-esculentus* system
LTi: tibia length
LTo: total hind leg length
MANOVA: multivariate analysis of variance
n. t.: new taxon
PCA: principal component analysis
PCR: polymerase chain reaction
PHR: peak height ratio
RAD: restriction site associated DNA
RAD-seq: restriction site associated DNA-sequencing
R-E system: *ridibundus-esculentus* system
SAI-1: *Serum-Albumin* intron 1
SNP: single nucleotide polymorphism
SVL: snout-vent length
