# Supplementary material for: Population genomics of an exceptional hybridogenetic system of Pelophylax water frogs
Source: BMC Evol Biol. 2019 Aug 5;19:164. doi: 10.1186/s12862-019-1482-4 (PMC6683362; doi:10.1186/s12862-019-1482-4)
Supplement: Supplementary file 1 — Figure S1. Average heterozygosity of Pelophylax populations mapped. (PDF 1249 kb) [file 12862_2019_1482_MOESM1_ESM.pdf]

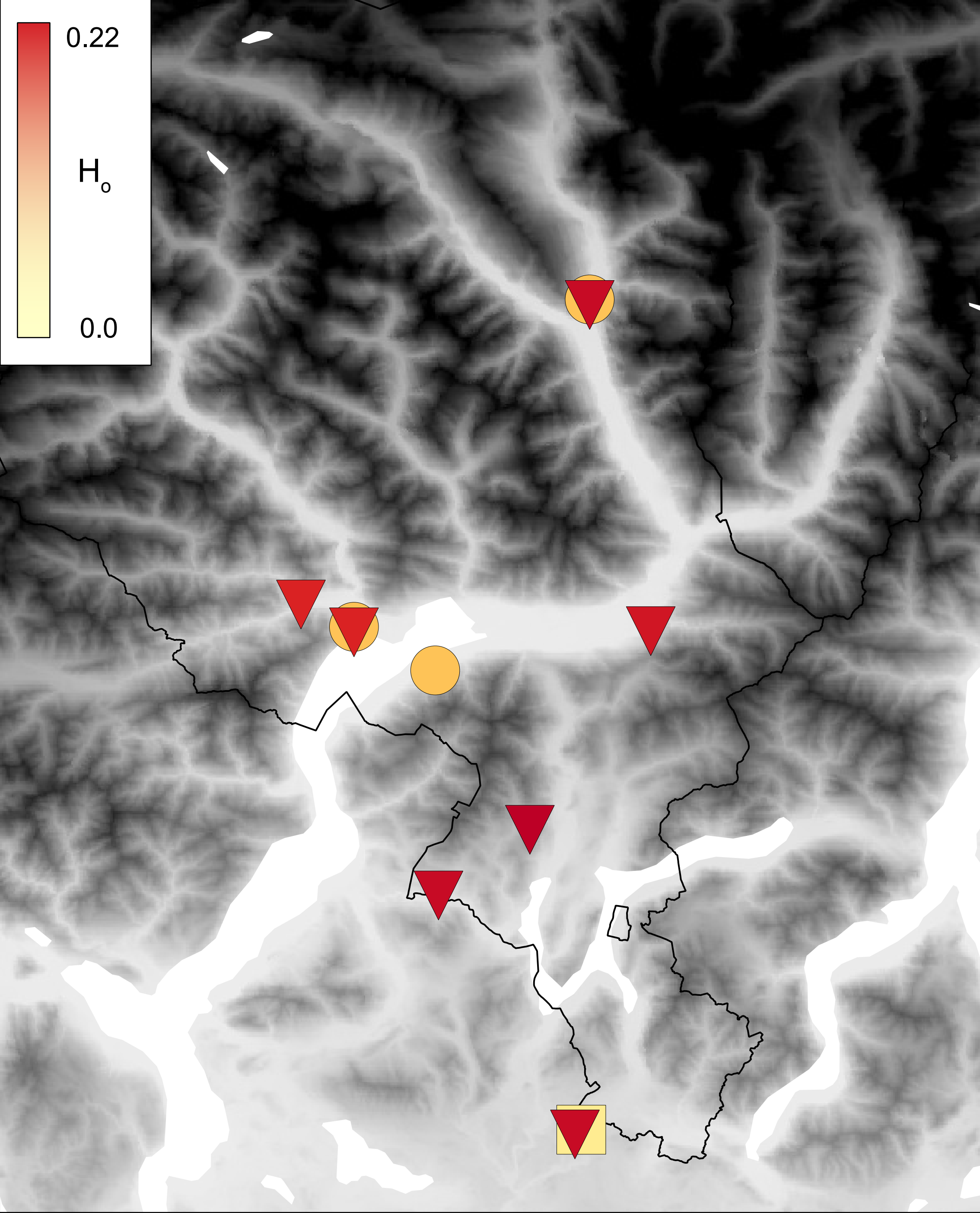

Fig. S1: Average heterozygosity of *Pelophylax* populations from Ticino, based on 2,521 SNPs. Circles: *P. lessonae*; triangles: *P.* kl. *esculentus*; square: *P. ridibundus*
